# Identification of the toxic 6mer seed consensus for human cancer cells

**DOI:** 10.1038/s41598-022-09051-w

**Published:** 2022-03-24

**Authors:** Monal Patel, Elizabeth T. Bartom, Bidur Paudel, Masha Kocherginsky, Kaitlyn L. O’Shea, Andrea E. Murmann, Marcus E. Peter

**Affiliations:** 1 Department of Medicine/Division of Hematology/Oncology, Feinberg School of Medicine, Northwestern University, Chicago, IL USA; 2 Department of Biochemistry and Molecular Genetics, Feinberg School of Medicine, Northwestern University, Chicago, IL USA; 3 Department of Preventive Medicine/Division of Biostatistics, Feinberg School of Medicine, Northwestern University, Chicago, IL USA

**Keywords:** Cell death, Cancer, Cell biology

## Abstract

6mer seed toxicity is a novel cell death mechanism that kills cancer cells by triggering death induced by survival gene elimination (DISE). It is based on si- or shRNAs with a specific G-rich nucleotide composition in position 2–7 of their guide strand. An arrayed screen of 4096 6mer seeds on two human and two mouse cell lines identified G-rich 6mers as the most toxic seeds. We have now tested two additional cell lines, one human and one mouse, identifying the GGGGGC consensus as the most toxic average 6mer seed for human cancer cells while slightly less significant for mouse cancer cells. RNA Seq and bioinformatics analyses suggested that an siRNA containing the GGGGGC seed (siGGGGGC) is toxic to cancer cells by targeting GCCCCC seed matches located predominantly in the 3′ UTR of a set of genes critical for cell survival. We have identified several genes targeted by this seed and demonstrate direct and specific targeting of GCCCCC seed matches, which is attenuated upon mutation of the GCCCCC seed matches in these 3′ UTRs. Our data show that siGGGGGC kills cancer cells through its miRNA-like activity and points at artificial miRNAs, si- or shRNAs containing this seed as a potential new cancer therapeutics.

## Introduction

micro(mi)RNAs are small noncoding double stranded RNAs that negatively regulate gene expression at the post-transcriptional level. Activity of miRNAs is determined by the seed sequence (position 2–7/8) in the guide strand of the miRNA^1,2^. The guide strand is incorporated into the RNA induce silencing complex (RISC), where it engages the targeted mRNA by binding to seed matches located mostly in the 3′ untranslated region (UTR) that are complementary to the miRNA's seed^3^. The human genome is estimated to contain up to 8300 miRNAs of which roughly 2300 have been confirmed^4^. They predominantly regulate differentiation and development and have been shown to be deregulated in virtually all human cancers, where they can function as tumor suppressors or oncogenes (oncomiRs)^5–7^.

We previously discovered that a large number of si- and shRNAs were toxic to all cancer cells independent of hitting their intended target^8^, in a process believed to be a special form of off-target effect of RNA interference (RNAi)^9^. It involves simultaneous activation of multiple cell death pathways^8^ and cancer cells cannot become resistant to this form of cell death. For an si- or shRNA to be toxic, a 6mer seed is sufficient^10^. We demonstrated that Ago2, a critical component of the RISC, was required for cells to die through 6mer seed toxicity (6mer Seed Tox), and that the toxic si/shRNAs acted like miRNAs by targeting the 3′ UTRs of essential survival genes^10^. The resulting form of cell death was therefore called DISE (for death induced by survival gene elimination)^11^. DISE was recently independently confirmed in the context of prostate cancer^12^. An arrayed screen of all possible 4096 6mer seeds in a neutral 19mer siRNA on two human and two mouse cell lines identified the most toxic seeds and a strong conservation across tissues and species. The most toxic 6mer seeds were high in G nucleotides at the 5′ end of the seed^13^. Consistent with a previous study that reported that most highly expressed genes regulating cell survival are devoid of seed matches for major miRNAs^14^, we found that survival genes are significantly enriched in potential C-rich seed matches in their 3′ UTR^13^. This suggested that while in the context of si/shRNAs that are designed to selectively silence single genes DISE could be viewed as an off-target effect, however, in the context of the biological function of miRNAs, this activity should be considered an on-target activity. In agreement with this interpretation are the findings that established major tumor suppressive miRNA families such as miR-34a,b,c,449a/b-5p and miR-15a/b,16-5p kill cancer cells largely through 6mer Seed Tox by targeting hundreds of survival genes^13,15,16^. si/shRNAs with a toxic 6mer seed can be used to treat cancer in mice; we demonstrated this in xenografted ovarian cancer without eliciting damage to normal tissues^17^.

The average toxic 6mer seed consensus in the top 200 most toxic seeds we described was GGGGGC^13^. We now demonstrate that an siRNA containing this consensus toxic seed motif (siGGGGGC) kills cancer cells indeed by targeting GCCCCC seed matches located predominantly in the 3′ UTR of abundant mRNAs, a number of which code for essential survival genes. Using an eCDF plot, we show a significant shift in the downregulated genes containing GCCCCC seed matches. In addition, we identify the most downregulated genes, many of which contain multiple GCCCCC seed matches in their 3′ UTR and demonstrate that silencing the ten most downregulated and highly expressed genes, using an siRNA SmartPool, induces cell death in transfected cells. Furthermore, we mutated GCCCCC seed matches in selected targeted genes and show that they cannot be efficiently silenced anymore by siGGGGGC when their 3′ UTRs were fused to a luciferase reporter gene. Finally, we confirm targeting of selected genes by demonstrating direct binding of siGGGGGC to its targets in a pull-down experiment of biotinylated siRNAs. We show that while the rules of targeting and toxicity apply to multiple cancer cells, the nature of targeted genes varies between cancer cells suggesting that the transcriptome of any cancer cell will likely always contain targetable GCCCCC seed matches in its expressed genes including in survival genes.

Our data strongly suggest that 6mer Seed Tox is an on-target mechanism regulating cell fate. When comparing the data of the siRNA screen between two human and two murine cell lines, we noticed a subtle difference in the composition of the toxic 6mer seed consensus (6merdb.org). We have now screened two more cell lines, one human and one mouse, and found that while the 100 most toxic 6mer seeds in human cells indeed on average contain the GGGGGC consensus, the three mouse cells are slightly less susceptible to high G containing siRNAs when compared to the three human cell lines. However, because siGGGGGC is still highly toxic to mouse cancer cells, it should therefore be feasible to test toxic 6mer seed containing si/shRNAs such as siGGGGGC in preclinical mouse models.

## Results and discussion

### The most toxic 6mer seeds differ slightly between human and mouse

**Figure 1:**
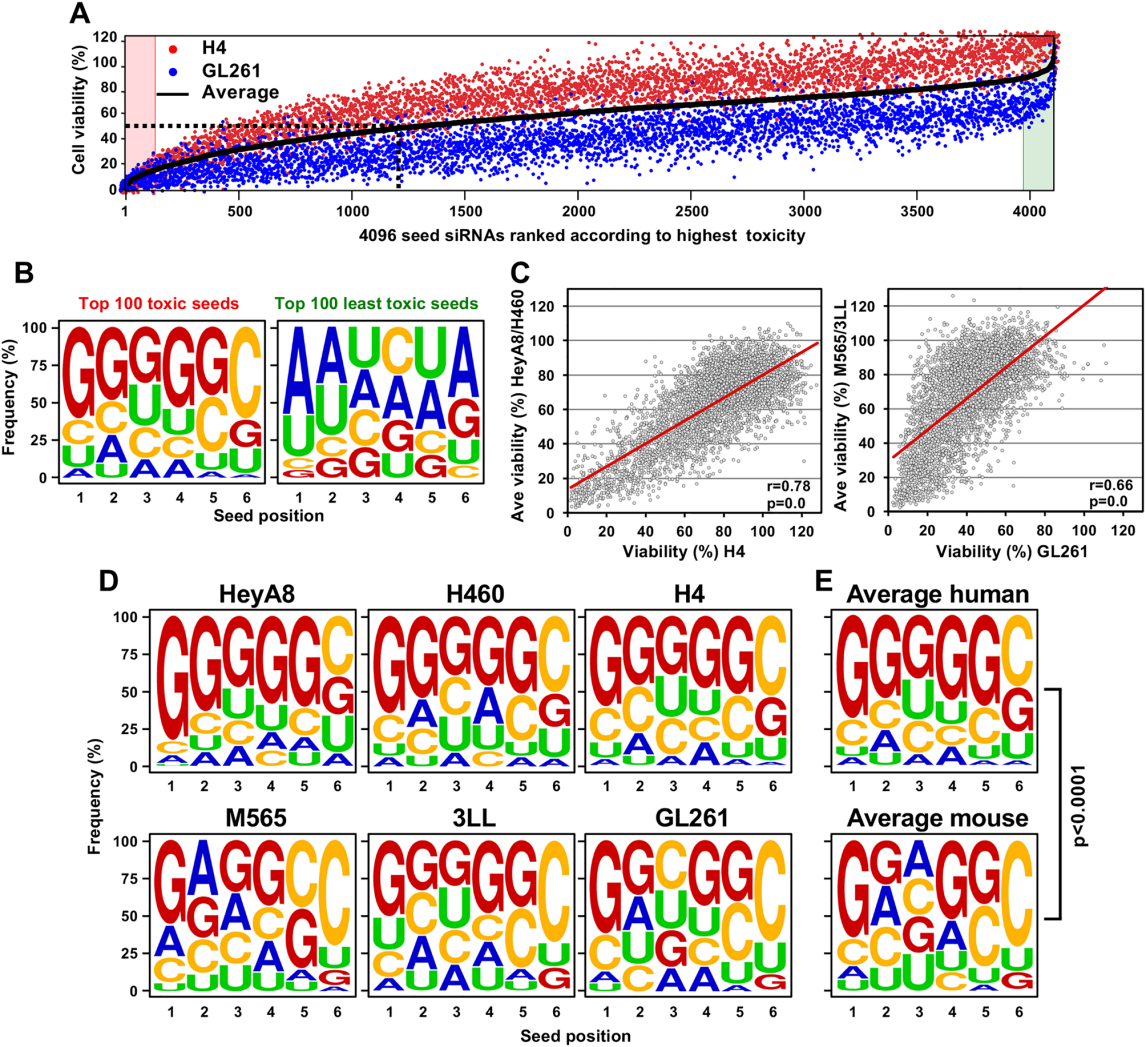
Subtle difference in the composition of the most toxic 6mer seed between human and mouse cancer cell lines. (**A**) Results of the 4096 6mer seed duplex screen in a human (H4) and a mouse (GL261) glioma cell line. Cells were reverse transfected in triplicates in 384 well plates with 10 nM of individual siRNAs. The cell viability of each 6mer seed duplex was determined by quantifying cellular ATP content 96 h after transfection. All 4096 6mer seeds are ranked by the average effect on cell viability of the two cell lines from the most toxic (left) to the least toxic (right). We consider an siRNA highly toxic if it reduces cell viability 90% or more and moderately toxic if it reduces cell viability 50% or more (black stippled line). (**B**) Average seed composition of the 100 most (left) and 100 least (right) toxic 6mer seeds in H4 and GL261 cells screened with 4096 6mer seed containing siRNAs. (**C**) *Left:* Regression analysis showing correlation between the 6mer Seed Tox observed in H4 cells and the average of the two previously tested human cell lines HeyA8 and H460. *Right:* Regression analysis showing correlation between the 6mer Seed Tox observed in GL261 cells and the average of the two previously tested murine cell lines 3LL and M565. p-values were calculated using Pearson correlation analysis. (**D**) Seed composition of the 100 most toxic 6mer seeds in three human (top) and three murine (bottom) cell lines. (**E**) Average most toxic seed composition of all human (top) and all murine (bottom) cell lines. p-value is derived from a multinomial mixed effects regression model.

Our recent arrayed screens of siRNAs containing all 4096 6mer seeds embedded in a neutral 19mer siRNA in two human cancer cell lines (HeyA8, ovarian and H460, lung cancer) as well as in two murine cell lines (M565, liver and 3LL, lung cancer) suggested that irrespective of tissue of origin or species, the most toxic 6mer seeds were rich in G, particularly at the 5′ end of the seed^13^. Minor differences were seen between the two human and two mouse cell lines. To determine whether this holds true when more cell lines representing additional tissues are tested, we screened all 4096 siRNAs on two additional cell lines, both with brain as the tissue of origin: the human neuroglioma cell line H4 and the murine glioma cell line GL261 (Fig. 1A). When plotting the average nucleotide composition of the 6mer seed of the 100 most toxic and 100 least toxic seeds of both cell lines, the results appeared to be very similar to the ones we had obtained previously for the four other cell lines, that is, again the most toxic seeds were high in Gs at the 5′ end of the seed, and the least toxic seeds were AU-rich with an A or U in the first two seed positions (Fig. 1B). The similarities between cell lines were also seen in a Pearson correlation analysis of the entire screen comparing each of the glioma cell line results with the average of the two previously analyzed cell lines from the same species (Fig. 1C). However, when assessing the average seed composition in the topmost toxic seeds for all 6 cell lines, subtle differences between human and mouse cells became apparent. The average seed composition of the most toxic seeds was more strongly dominated by Gs in the three human cells compared to the three mouse cell lines (Fig. 1D,E). These sequence logos (generated with Weblogo) are a graphical representation of the relative frequency of each nucleotide in each of the 6 positions of the 6mer seed. Existing sequence logo methods only permit a visual representation. We therefore developed a framework for statistical analysis of 6mer toxicity data using multinomial mixed effects regression models. This approach allows us to compare differences in sequence patterns between groups and positions and provides both a statistical testing framework for comparing nucleotide composition between groups and positions, as well as estimates of the differences. Applying this new approach, we found that there were statistically significant differences in nucleotide patterns between human and mouse composition of the top 100 most toxic seeds (p < 0.0001) (Fig. 1E). Compared to G, nucleotides A (odds ratio OR = 2.49, p < 0.0001), C (OR = 1.96; p < 0.0001) and U (OR = 1.51; p = 0.020) were more likely to occur in mice than in humans. The differences between groups did not vary by position (p = 0.245, group × position interaction term). The difference in toxic seed composition was also visible when all 4096 seeds were ranked according to the highest average toxicity in the human cells (note, the different colors between the three human and the three mouse cell lines in Table S1). The siRNA harboring the average consensus GGGGGC was the 8th most toxic seed for human cells but only the 110th most toxic seed for the three mouse cell lines (marked in a red font in Table S1). These data have now been added to the 6merdb.org site. They suggest that while G-rich 6mer seeds are highly toxic to both human and mouse cells, the very high toxicity of G rich seeds is most prominent in human cells. In human cells the average most toxic seed consensus is GGGGGC.

### siGGGGGC is highly toxic to multiple cancer cells

**Figure 2:**
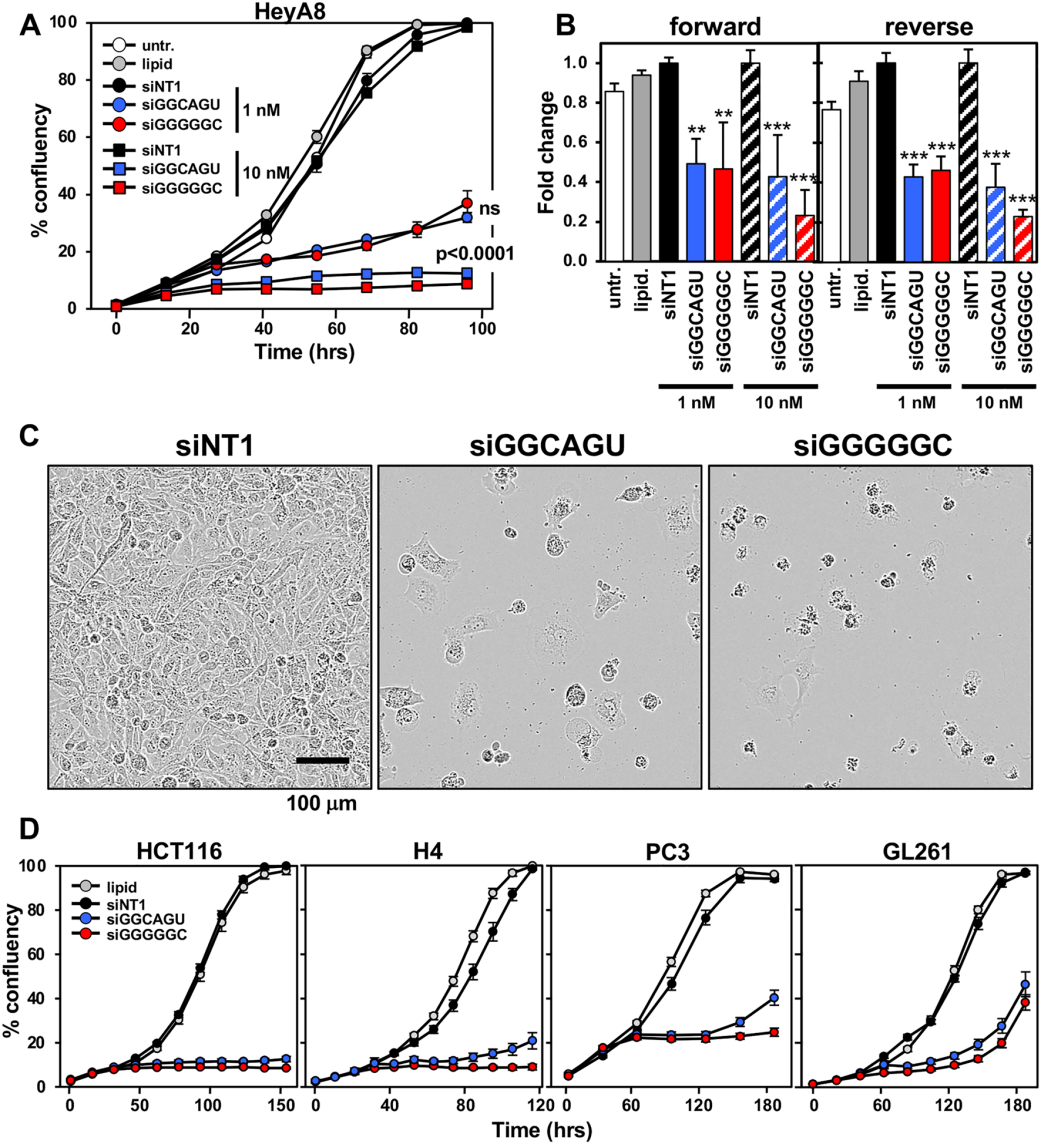
Both siGGCAGU and siGGGGGC are toxic to cancer cells. (**A**) Change in confluence (growth) over time of HeyA8 cells reverse transfected with either 1 or 10 nM of siNT1, siGGCAGU, or siGGGGGC. ANOVA p-values between the two toxic siRNAs are shown. *ns* not significant. Each data point represents mean ± SE of six replicates. (**B**) Percent viability (ATP content) 96 h after transfecting HeyA8 cells (forward or reverse) with 1 or 10 nM of the indicated siRNAs, or with lipid only or left untreated. Experiment was done in 6 replicates. ***p < 0.0001, **p < 0.001. (**C**) Phase contrast images showing morphology of HeyA8 cells reverse transfected in A at 10 nM 4 days post transfection. (**D**) Change in confluence over time of HCT116 (human colon cancer) and H4 (human neuroglioma), PC3 (human prostate cancer), or GL261 (mouse glioma) cells reverse transfected with 10 nM (HCT116, H4, and PC3) or 25 nM (GL261) of siNT1, siGGCAGU, or siGGGGGC. Each data point represents mean ± SE of six or four replicates, respectively. In all three cases the optimal amount of transfection reagent (lipid) was used to avoid non-specific growth inhibition.

We recently reported that the prototypical tumor suppressor miRNA, miR-34a-5p, is toxic to various cancer cells mostly through 6mer Seed Tox by targeting the 3′ UTR of multiple genes that are critical for the cell survival^13^. miR-34a-5p carries two Gs as the first two nucleotides in its 6mer seed GGCAGU, consistent with the observation of a high G content towards the 5′ end of the most toxic seeds. We have now compared the potency of siGGCAGU to siGGGGGC, the seed of which is present among other miRNAs, in miR-1237-5p. Both siRNAs had a chemically modified passenger strand to prevent loading into the RISC^13,18^. When transfected into the ovarian cancer (OC) cell line HeyA8, both siRNAs strongly slowed down cell growth (Fig. 2A) and reduced cell viability compared to the nontoxic siNT1, regardless of whether the siRNAs were introduced into cells by forward or reverse transfection (Fig. 2B). Morphologically, cell death was very similar to what we had reported for cells undergoing DISE (Fig. 2C)^8^. Our previous data suggested that DISE is causing a strong stress response in cells resulting in ROS production and DNA damage^8^. Consistently, cells transfected with siGGGGGC show signs of severe stress with large intracellular vesicles forming before the onset of cell death^8^ (Movies S1–S3). In all tested human and murine cell lines, siGGGGGC was slightly more toxic than siGGCAGU (Fig. 2D).

### Both siGGGGGC and siGGCAGU induced cell death is similar to DISE and acts by targeting mRNAs in a miRNA-like fashion

**Figure 3:**
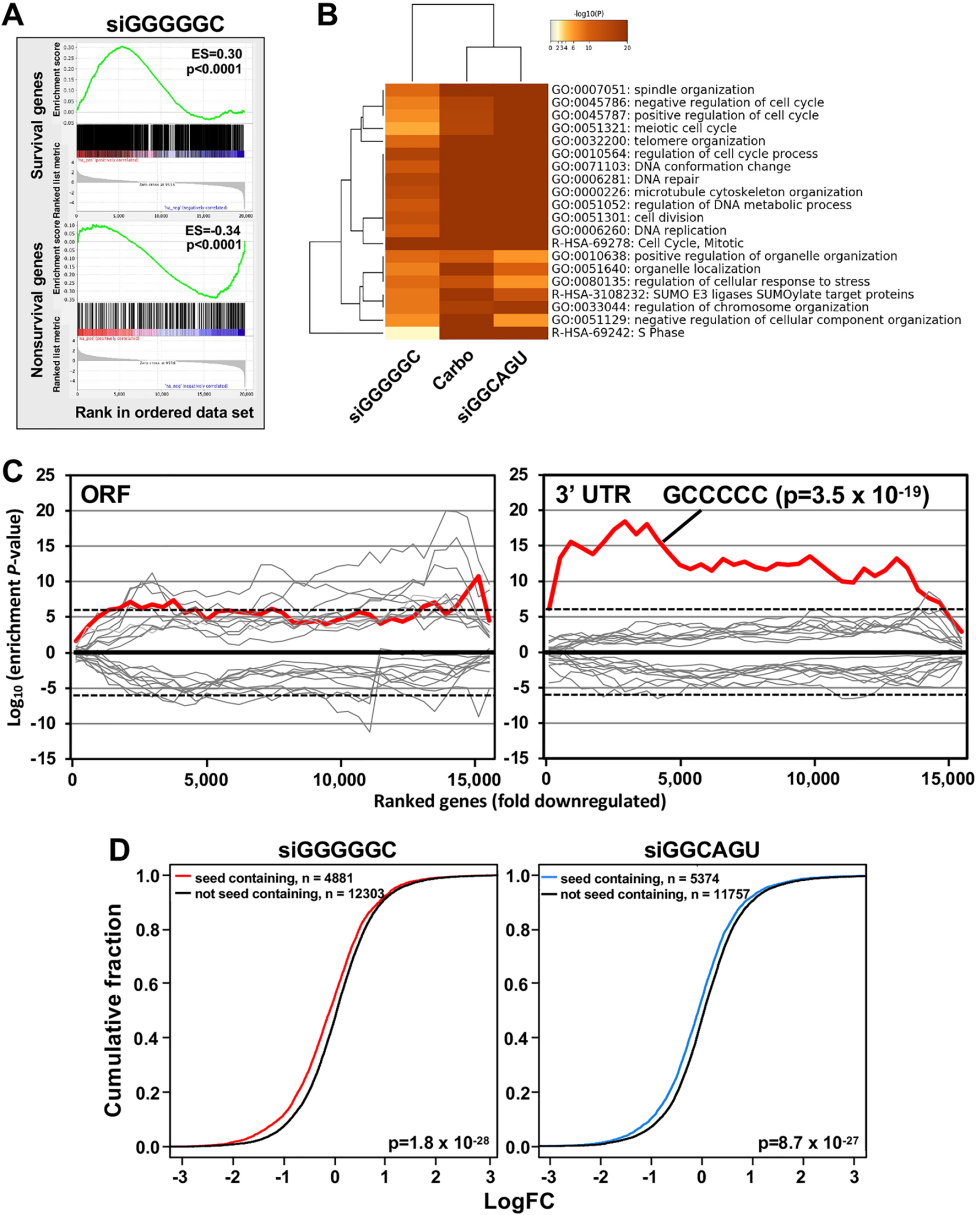
siGGGGGC is toxic to cancer cells through DISE induction. (**A**) Gene set enrichment analysis for a group of 1846 survival genes (top panel) and 416 non-survival genes (bottom panel)^10^ 48 h after transfecting HeyA8 cells with siGGGGGC. siNT1 served as a control. p-values indicate significance of enrichment; the enrichment score (ES) is shown. (**B**) Metascape gene ontology analysis comparing genes significantly downregulated from the RNA-seq data in HeyA8 cells treated with siGGGGGC, carboplatin or siGGCAGU for 48 h. (**C**) Sylamer analysis (6mers) for the list of open reading frames (ORFs; left) and 3′ UTRs (right) of mRNAs in cells treated with 10 nM siGGGGGC for 24 h sorted from down-regulated to up-regulated. The 10 most and ten least enriched seeds are shown. Enrichment of the GCCCCC sequence is shown in red, other sequences are in grey. The stippled line corresponds to a p-value threshold of 0.05 after Bonferroni correction for the number of words tested (4096). Bonferroni-adjusted p-value is shown. (**D**) eCDF plots of mRNAs (in RNA isolated from PC3 cells 24 and 48 h after transfection) containing a unique 6mer seed match for either siGGCAGU (left) or siGGGGGC (right) in their 3′ UTR vs those that do not. The p-values were calculated using a two-sample Kolmogorov–Smirnov (K–S) test (alternative hypothesis = “greater”).

To determine how siGGGGGC induces cell death, we performed an RNA Seq analysis of HeyA8 cells at both 24 and 48 h after transfection with 10 nM of siNT1 or siGGGGGC. The genes downregulated in the siGGGGGC transfected cells 48 h after transfection were enriched in a reference set of recently defined survival genes^10^ (Fig. 3A), similar to the activity we reported for the two tumor suppressive miRNAs miR-34a-5p and miR-15/16-3p and their matching 6mer seed containing siRNAs^13,15^. A gene ontology analysis suggested that the form of cell death induced by siGGGGGC, siGGCAGU, and the chemotherapeutic drug carboplatin were very similar with shared GO terms we had previously reported to characterize DISE (Fig. 3B)^10,13,19^. This finding is supported by our recent report that carboplatin indeed kills OC cells in part through 6mer seed toxicity and that platinum resistant OC cells are hypersensitive to both siGGGGGC and siGGCAGU^20^. To determine whether siGGGGGC targets mRNAs that contain GCCCCC 6mer seed matches in their 3′ UTR, we analyzed mRNAs deregulated in HeyA8 cells 24 h after transfection with siGGGGGC (Fig. 3C). We chose the 24 h time point for this analysis to only detect early changes in mRNA levels. Using a Sylamer analysis as a method to identify enriched sequences in a list of downregulated mRNAs^21^, we found a strong enrichment of the GCCCCC sequence expected to be targeted by siGGGGGC in the 3′ UTR of downregulated mRNAs (Fig. 3C, right) but not in their ORF (Fig. 3C, left). An eCDF plot, an established form of analysis to identify target engagement by miRNAs, confirmed the targeting of multiple mRNAs by siGGGGGC (Fig. 3D); the result of a similar analysis in cells transfected with siGGCAGU is shown for comparison. This analysis suggested that siGGGGGC induced cell death by targeting GCCCCC seed matches present predominantly in the 3′ UTR of genes.

### siGGGGGC acts like a miRNA by targeting GCCCCC seed matches enriched in the 3′ UTR of several survival genes

**Figure 4:**
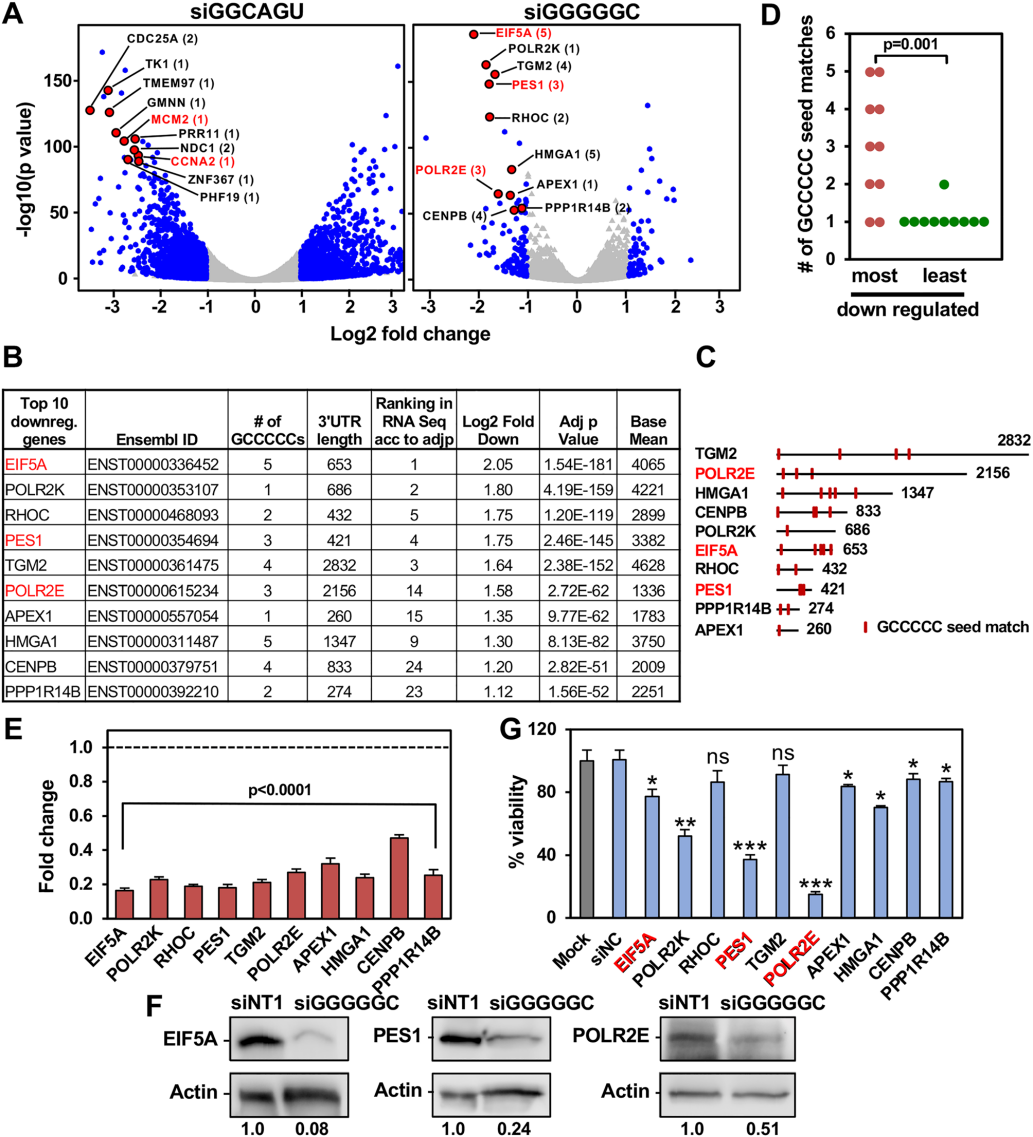
siGGGGGC kills cells by targeting GCCCCC seed matches located predominantly in the 3′ UTR of targeted mRNAs. (**A**) Volcano plot of deregulated genes in HeyA8 cells transfected with 10 nM siGGGGGC vs. siNT1 (after 24 h) or with 10 nM siGGCAGU vs. siNT1 (after 48 h). Red dots indicate genes that were most highly expressed (>1000 norm reads) and down regulated. The number of GCCCCC seed matches is given in brackets. Grey dots indicate all differentially expressed genes (with a log2 fold change < 3 and > − 3), blue dots indicate genes with log2 fold change > 1 and FDRAdjpValue < 0.05. (**B**) Table showing the 10 downregulated genes labeled in A ranked (from top to bottom) according to Log2 fold change. (**C**) Schematic of the 3′ UTRs of the 10 genes listed in B with the location of the GCCCCC seed matches. (**D**) The number of predicted seed matches in the ten most and least downregulated and highly expressed genes part of the 5957 genes in the human genome that contain at least one such seed match in their 3′ UTR. p-value was calculated by Student's *t* test. (**E**) Real-time PCR validation of downregulation of the ten genes in (**B**) derived from RNA seq analysis (24 h time point). Gene expression levels in HeyA8 cells transfected with siGGGGGC were normalized to GAPDH and to cells transfected with siNT1 (stippled line). Each bar represents ± SD of three replicates. Student’s *t* test was used to calculate p-values for each gene. (**F**) Western blot analysis for EIF5A, POLR2E, PES1 and actin in HeyA8 cells transfected with 20 nM siNT1 or siGGGGGC for 48 h. Quantification of bands normalized to actin is shown. (**G**) Viability of HeyA8 cells 96 h after transfection with 10 nM of siRNA SmartPools against the ten genes in B. Each bar represents ± SD of three replicates. Student’s *t* test, ***p < 0.0001, **p < 0.001, *p < 0.05, ns, not significant. Genes that are part of our curated list of survival genes are labeled in red.

**Figure 5:**
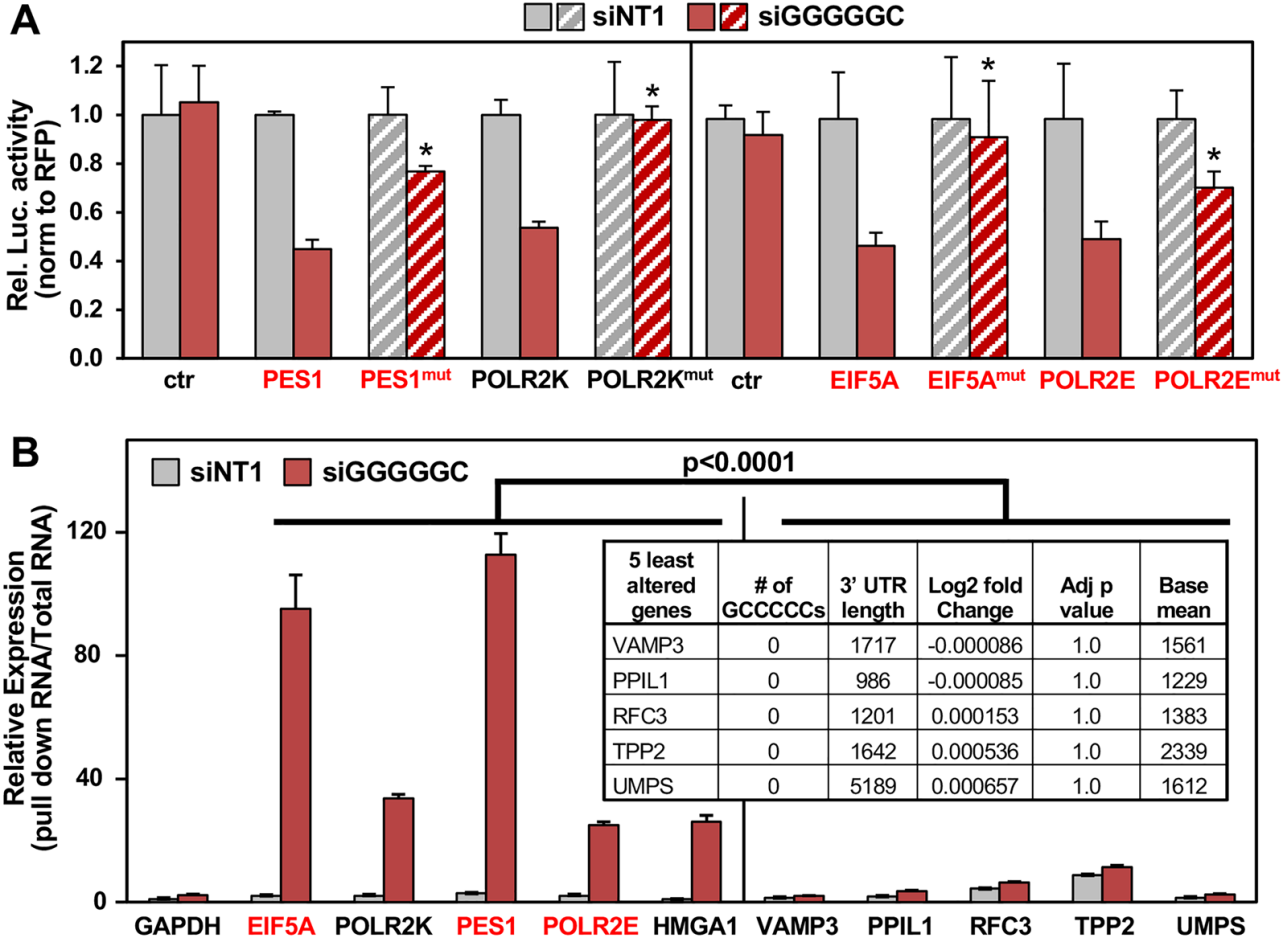
Validation of GCCCCC seed matches located in the 3′ UTR of abundant genes being targeted by siGGGGGC. (**A**) Relative luciferase activity of control plasmid (pmiR-Target), wild-type and mutant 3′ UTR constructs of 4 of the genes in Fig. 4B in 293T cells. Data are shown as mean ± SD of three replicates. p-values (Student’s *t* test, *p < 0.05) represent comparison between change in relative luciferase of cells co-transfected with wt/mutant constructs (50 ng) and siGGGGGC (20 nM) normalized to cells transfected with siNT1. (**B**) Enrichment of siGGGGGC target mRNAs in biotin-streptavidin pulled down RNA normalized to input RNA in HeyA8 cells transfected with 50 nM Bi-siNT1 or Bi-siGGGGGC for 20 h by real-time PCR. Inset: Table showing 5 selected control genes (> 1000 normReads) expression of which did not change in RNA-seq data derived from HeyA8 cells transfected with 10 nM siGGGGGC vs. siNT1 for 24 h. Each bar represents ± SD of three replicates. Student’s *t* test was used to calculate p-value. Survival genes are shown in red.

We previously provided evidence that siGGCAGU targets mRNAs that carry the miR-34a-5p seed match^13^. Indeed, a volcano plot of RNA isolated from cells 48 h after transfection confirmed that most of the highly abundant and most highly downregulated genes (top ten are shown as red dots in Fig. 4A, left panel) carry at least one ACUGCC seed match in their 3′ UTR and two of the top 10 were part of the curated list of survival genes (labeled red in Fig. 4A, left and Table S2). To determine whether GCCCCC seed matches were indeed also being targeted by siGGGGGC in a miRNA-like fashion, we identified the 10 most significantly downregulated and highly (base mean > 1000) expressed mRNAs that contain at least one putative GCCCCC sequence in their 3′ UTR (Fig. 4A, right panel and Table S2). We chose the 24 h time point for this analysis as siGGGGGC is more toxic than siGGCAGU and we were interested in early deregulated genes in this analysis. Of these ten genes, three were part of our curated list of survival genes (labeled red in Fig. 4A, right). Most of them contained multiple GCCCCC sequences in their 3′ UTRs (Fig. 4B,C). In fact, these ten most downregulated genes (out of 304 genes) contained significantly more seed matches than the ten least downregulated genes (Fig. 4D and Table S2). We confirmed by qPCR that all ten genes were effectively silenced in the cells transfected with siGGGGGC (Fig. 4E) and the three survival genes were also confirmed by Western blotting of cells 24 h after transfection with siGGGGGC (Fig. 4F). When these ten mRNAs were then targeted using specific SmartPools of four siRNAs each, eight of them significantly reduced cell viability, with three of the siRNA pools being most toxic (Fig. 4G), two of which targeted established survival genes (PES1 and POLR2E). These data support our conclusion that siGGGGGC is toxic to cancer cells by silencing GCCCCC seed match containing genes that are critical for cell survival. To confirm that siGGGGGC was indeed specifically targeting GCCCCC seed matches, we cotransfected cells with siGGGGGC and a luciferase reporter construct carrying either the wt 3′ UTR or a version with mutated GCCCCC seed matches of four of the genes we found to be important for cell survival in HeyA8 cells (PES1, POLR2K, EIF5A, and POLR2E) (Fig. 5A). In each case, the mutant 3′ UTR construct was more resistant to the suppressive activity of siGGGGGC than the wt construct. Finally, we pulled down biotinylated (Bi-)siGGGGGC transfected into HeyA8 cells and demonstrated that some of the key downregulated GCCCCC seed match containing genes were indeed associated with the transfected siGGGGGC (Fig. 5B). Confirming specificity, 5 genes with comparable expression levels that do not contain a GCCCCC motive and that were not silenced did not specifically associate with siGGGGGC. In summary, our data indicate that siRNAs with toxic 6mer seeds act like miRNAs by targeting 6mer seed matches located predominantly in the 3′ UTR of targeted genes and that small amounts of these siRNAs are sufficient to kill cells.

### The network of targeted GCCCCC seed match containing genes differs between different cancer cells

Given the nature of the toxicity of siGGGGGC, we would expect it to kill all cancer cells through the same general mechanism: targeting a network of genes containing GCCCCC seed matches in their 3′ UTR and many of these genes might be survival genes. We found that we could kill multiple cancer cell lines using siGGGGGC (see Fig. 2D and data not shown). To demonstrate that this mechanism of toxicity also applied to another cell line we chose the prostate cancer (PC) cell line PC3 as the DISE concept was previously confirmed for PC^12^. Cells were transfected with 10 nM siNT1 or siGGGGGC and after 24 and 48 h subjected to an RNA-Seq analysis. Similar to the results with the OC cell line HeyA8, downregulated genes in siGGGGGC transfected PC3 cells were enriched in survival genes (Fig. S1A). A Sylamer analysis confirmed targeting of GCCCCC seed matches in the 3′ UTR of downregulated genes (Fig. S1B), and this enrichment was also seen in an eCDF plot (Fig. S1C). Interestingly, in PC3 cells there seemed to be a significant enrichment of GCCCCC sequences also in the ORF (second most highly enriched 6mer), however, a number of other 6mer seed matches were equally enriched suggesting that these were unspecific events. To determine which highly expressed genes are being targeted in PC3 cells, we again identified the 10 genes with normCounts > 1000 that were most highly downregulated in the siGGGGGC treated versus the siNT1 treated cells (Fig. S1D). The majority of these genes had multiple GCCCCC sequences in their 3′ UTR. The top five most abundant of these genes were significantly downregulated 24 h after transfection and silencing was even more pronounced 48 h after transfection (Fig. S1E). The silencing of these genes was also confirmed in an independent experiment using real time PCR (Fig. S1F). A volcano plot showed the degree of significantly up- and downregulated genes in the siGGGGGC treated cells 24 h after transfection (Fig. S1G). Interestingly, the top five most downregulated and abundant GCCCCC containing genes in PC3 cells transfected with siGGGGGC was different from the ones downregulated in HeyA8 cells (Fig. S1G). This raised the question of how many highly expressed genes would be equally targeted in both cell lines. To directly compare targeting in the two cell lines, we plotted the expression levels of all highly expressed genes carrying a GCCCCC seed match in their 3′ UTRs determined by an RNA-Seq analysis of Mock treated PC3 and HeyA8 cells (Fig. S1H). While there were substantial similarities between the two GCCCCC containing transcriptomes, there were also significant differences overall. However, expression levels of survival genes were more similar in both cells (r = 0.96) than of all genes (r = 0.78). The data suggest that there will likely always be enough highly expressed genes containing GCCCCC sequences to be targeted by a GGGGGC 6mer seed containing short RNAs and enough survival genes to be targeted to kill most if not all cancer cells.

6mer Seed Tox is an intrinsic anti-cancer mechanism possibly built into all genomes of multicellular organisms that harbor miRNAs. It is based on the understanding that highly expressed miRNAs must not be toxic to normal cells. Hence, 3′ UTRs of genes critical for cell survival are generally devoid of seed matches for abundant miRNAs^14^. Consistent with this observation, we reported that older and more conserved miRNAs contain fewer toxic 6mer seeds and that many younger miRNAs, including most miRtrons, which have G-rich seeds are barely expressed in cells^13^. Based on an analysis of young and old miRNAs, we concluded that the 6mer Seed Tox/DISE mechanism is at least 800 million years old. By testing two more cell lines, one human and one mouse, we now provide evidence that the G-rich toxic mechanism is conserved between human and mouse cells with only minor differences in the most toxic seeds possibly pointing at ongoing evolutionary development. However, this would need to be confirmed by testing larger numbers of cell lines, including other species.

We have now identified several direct targets of siGGGGGC in two cancer cell lines representing ovarian and prostate cancer, respectively, which are strongly silenced in the cells shortly after transfection. In principle, these targets could be used as biomarkers for silencing to confirm target engagement during a potential treatment of cancer with this toxic seed containing RNA oligonucleotide. However, there can be no definite list of targeted genes for all cancers or cancer cells, as the nature of the targeted genes will likely depend on the transcriptome of every cancer, and due to intratumor heterogeneity of every individual cancer cell. In vivo targeting can be established by performing RNA Seq of tumor tissue after treatment and subjecting the data to the bioinformatics analyses as presented here. In addition, targeted genes containing predicted GCCCCC seed matches, that could serve as biomarkers in patient tumors, could be identified on a single cell level using single cell RNA-Seq.

We have previously shown that siRNAs that are toxic through 6mer Seed Tox can be used to treat OC in mice without any signs of toxicity to the mice^17^. While some normal cells when cultured on plastic (e.g. human keratinocytes) grow as rapidly as cancer cells and become sensitive to DISE (unpublished data), our own analysis of immortalized ovarian fibroblasts^8^, and a recent study comparing prostate cancer cells and normal prostate epithelium^12^, suggested that many normal cells are less susceptible to DISE than cancer cells. The reason for this selectivity, particularly in vivo*,* is currently being investigated.

Using the 6mer seed of an established tumor suppressive miRNA such as miR-34a-5p, which has already been tested in a clinical trial for the treatment of various human cancers^22^, may be a viable new treatment option for advanced cancers, particularly as we recently demonstrated that OC cells resistant to platinum, the first line treatment for OC, show an increased sensitivity to 6mer seed toxicity^20^. However, rather than using the entire miRNA, our data suggest that it may be sufficient to use a short RNA only containing the toxic 6mer seed of a miRNA. Our analysis identifies the 6mer seed GGGGGC as the average consensus sequence for the most toxic 6mer seed for human cells. While there are subtle differences in the composition of the most toxic seeds for mouse cells, it should still be possible to test the GGGGGC seed containing si- or shRNAs in preclinical mouse tumor models as the GGGGGC seed is highly toxic to both human and mouse cancer cells.

## Materials and methods

### Cell lines

HeyA8 and PC3 cells were grown in RPMI 1640 medium (Corning #10-040 CM) with 10% heat-inactivated fetal bovine serum (FBS) (Sigma-Aldrich #14009C), 1% l-glutamine (Mediatech Inc), and 1% penicillin/streptomycin (Mediatech Inc). 293T, GL261 and H4 cells were cultured in DMEM medium (Corning #10-013-CM) supplemented with 10% FBS and 1% penicillin/streptomycin. HCT116 cells were cultured in McCoy’s 5A medium (ATCC #30-2007) supplemented with 10% FBS and 1% l-glutamine.

### Reagents

Lipofectamine RNAimax (#13778150) and Lipofectamine 2000 (#11668019) were from Thermofisher Scientific. Optimem was purchased from Gibco (#31985-070). Cell-Titer Glo was from Promega (#G7570). Western blot antibodies were from the following sources: anti-EIF5A (ABclonal #A2016); anti-PES1 (#PA564020) and anti-POLR2E (#MA525454) were purchased from Thermo Fisher Scientific; anti-β-actin antibody (#sc-47778) was purchased from Santa Cruz. The secondary antibodies goat anti-rabbit IgG-HRP (#4030-05) and goat anti-mouse IgG2a-HRP (#1081-05) were from Southern Biotech.

### Transfections and cell growth assessment

To assess cell growth over time using IncuCyte Zoom live-cell imaging system (Essen Bioscience), cells were either reverse or forward transfected in 96-well plates using Optimem containing optimized amounts of RNAimax and either 1 or 10 nM of siRNA. For IncuCyte experiments, the following plating densities were used per 96-well: HeyA8 (1000 cells), PC3 (3000 cells), HCT116 (2200 cells), H4 and GL261 (3000 cells). Growth curves were generated in Excel using percent confluence data over time obtained using IncuCyte Zoom software. Custom siRNA oligonucleotides were ordered from Integrated DNA Technologies and oligonucleotides were annealed according to the manufacturer’s instructions. The siRNAs containing the toxic seeds were designed as described previously^13^. The sequences used were as follows:

- siNT1 sense: mUmGrGrUrUrUrArCrArUrGrUrCrGrArCrUrArATT
- siNT1 antisense: rUrUrArGrUrCrGrArCrArUrGrUrArArArCrCrAAA
- siGGCAGU sense: mUmGrGrUrUrUrArCrArUrGrUrArCrUrGrCrCrATT
- siGGCAGU antisense: rUrGrGrCrArGrUrArCrArUrGrUrArArArCrCrAAA
- siGGGGGC sense: mUmGrGrUrUrUrArCrArUrGrUrGrCrCrCrCrCrATT
- siGGGGGC antisense: rUrGrGrGrGrGrCrArCrArUrGrUrArArArCrCrAAA

For knocking down the ten downregulated genes in Fig. 4F, HeyA8 cells were reverse transfected using Optimem and 0.1 μl RNAimax per 96-well in triplicates with 10 nM of either of the following ON-TARGETplus human siRNA SmartPools purchased from Dharmacon: EIF5A (#L-015739-00-0005), PES1 (#L-009542-00-0005), POLR2E (L-004739-01-0005), HMGA1 (#L-004597-00-0005), TGM2 (#L-004971-00-0005), POLR2K (#L-011979-01-0005), RHOC (#L-008555-00-0005), APEX1 (#L-010237-00-0005), CENPB (#L-003250-00-0005), PPP1R14B (#L-026574-00-0005). The Non-targeting control pool (#D-001810-10-05) was used as a negative control.

### siRNA screens and cell viability assay

The 4096 siRNAs were described and used in an arrayed screen as described previously (11). In brief, transfection efficiency was optimized for two more cell lines (H4 and GL261) individually. RNA duplexes were first diluted with Opti-MEM to make 20 μl solution of 10 nM (for H4 cells) and 25 nM (for GL261 cells) as final concentration in a 384‐well plate by Multidrop Combi. Lipofectamine RNAimax (Invitrogen) was diluted in Opti-MEM (5.8 μl lipid + 994.2 μl of Opti-MEM for H4, 52.6 μl lipid + 947.4 μl of Opti-MEM for GL261). After incubating at room temperature for 5–10 min, 20 μl of the diluted lipid was dispensed into each well of the plate containing RNA duplexes. The mixture was pipetted up and down three times by PerkinElmer EP3, incubated at room temperature for at least 20 min mixed again using a PerkinElmer EP3. 10 μl of the mixture was then transferred into wells of three new plates (triplicates) using the PerkinElmer EP3. 40 μl cell suspension containing 550 H4 or 550 GL261 cells was then added to each well containing the duplex and lipid mix resulting in a final volume of 50 μl. Plates were left at room temperature for 30 min and then moved to a 37 °C incubator. 96 h post transfection, cell viability was assayed using CellTiter‐Glo quantifying cellular ATP content. 25 μl medium was removed from each well, and 20 μl CellTiter‐Glo cell viability reagent was added. The plates were shaken for 5 min and incubated at room temperature for 15 min. Luminescence was then read on the BioTek Synergy Neo2. The 4096 6mer seed containing duplexes were screened in three sets for each cell line. Each set was comprised of five 384 well plates. A number of control siRNAs of known toxicity (including siNT1 and siL3^10^) was added to each plate to compare reproducibility. All samples were set up in triplicate (on three different plates = 15 plates/set). Screen results were normalized to the average viability of the cells to siNT1 correcting the variability between sets. For the viability assays in Figs. 2B and 4G, 96 h post transfection the medium in 96-wells was replaced with 70 μl of fresh media and 70 μl of CellTiter-Glo reagent was added on top and the luminescence was then measured as above using BioTek Cytation 5.

### RNA-seq analysis

For RNA-seq experiment 100,000 HeyA8 cells or 200,000 PC3 cells were reverse transfected with siGGGGGC, siGGCAGU or non-targeting siNT1 (siUAGUCG) duplex in multiple 6-well plates in duplicates, each well with 500 μl Optimem mix containing 1 μl RNAimax per well and siRNA at 10 nM plus 2 ml media per well. Cells were lysed the next day using Qiazol for the 24 h time point, and to the remaining wells media was changed at 24 h and then cells were lysed with Qiazol at 48 h post transfection. A DNAse digestion step was included using the RNAse-free DNAse set (Qiagen # 79254). Total RNA was then isolated using the miRNeasy Mini Kit (Qiagen # 74004). The quality of RNA was determined using an Agilent Bioanalyzer. The RNA library preparation and subsequent sequencing on Illumina HiSEQ4000 were done by Nu-Seq core at Northwestern University (Chicago). Paired end RNA-seq libraries were prepared using Illumina Tru-Seq Total RNA Library Prep Kit and included a RiboZero rRNA depletion. Reads were trimmed with Trimmomatic v0.33 (TRAILING:30 MINLEN:20)^23^ and then aligned to the hg38 human genome assembly with Tophat v2.1^24^ or STAR^25^. Exonic reads were assigned to genes using the Ensembl 78 version of the hg38 transcriptome and htseq v0.6.1^26^. Differential expression analysis was carried out using the edgeR package^27^ to fit a negative binomial generalized log-linear model to the read counts for each gene.

### Data analyses

GSEA was performed using the GSEA v4.0.3 software from the Broad Institute (https://software.broadinstitute.org/gsea/). From the RNA-seq data, a ranked list was generated by sorting genes according to the Log_10_(fold downregulation) and this list was then used to perform GSEA using the pre-ranked function and 1000 permutations were used. The survival and non-survival gene sets previously described^10^ were used as custom gene sets. The GO enrichment analysis was performed with all genes that after alignment and normalization were at least twofold downregulated with an adjusted p-value of < 0.05 using the software available on http://www.Metascape.org; default settings were used. The data set on carboplatin treated HeyA8 cells was previously published^13^, accession number: GSE111379.

Sylamer analysis was performed as recently described^13^. 3′ UTRs or ORFs were used from Ensembl, version 76.

Volcano plot was generated using RStudio. Using the list of genes differentially expressed in either HeyA8 cells transfected with 10 nM siGGGGGC or siGGCAGU vs. siNT1 (24 h) or PC3 cells transfected with 10 nM siGGGGGC vs. siNT1 (24 and 48 h combined) derived from the RNA-seq data, the R-script plotted all genes with a log2 fold change of < 3 and > − 3 on x-axis and − log_10_pValue on y-axis.

To identify the most significantly downregulated genes in cells transfected with siGGGGGC or siGGCAGU that carry at least one putative seed match GCCCCC or ACUGCC, respectively, in their 3′ UTR, we used a data set previously described with the number and location of all 6mer motifs in the 3′ UTR of all human genes^18^ (sequences extracted from ensembl.org). All genes differentially expressed (adjp < 0.05, > 1 Log2 fold deregulated) between cells transfected with siGGGGGC or siGGCAGU vs. siNT1 with a base mean of 1000 or higher were compared to the list of genes with the GCCCCC or ACUGCC seed matches, respectively. Transcripts ranked according to higher fold downregulation in the siGGGGGC and siGGCAGU transfected HeyA8 cells are shown in Table S2. The 5 genes that are not changed in transfected PC3 cells shown in Fig. 5B were identified by ranking all genes (> 1000 normReads) from highest to lowest fold change and then selecting the 5 genes with the least change. The top most highly downregulated and bottom least downregulated transcripts in siGGGGGC transfected HeyA8 cells were used to generate Fig. 4D.

### Real-time PCR

Real-time PCR was performed for the top 10 most downregulated and highly expressed genes in HeyA8 cells (Fig. 4B) and the 5 most downregulated and highly expressed genes in PC3 cells (Fig. S1D). Briefly, 200 ng total RNA was used to make cDNA using the High-Capacity cDNA Reverse Transcription Kit (Applied Biosystems #4368814). The qPCR was then done using the Taqman Gene Expression Master Mix (ThermoFisher Scientific #4369016) and the following primers (human) from ThermoFisher Scientific: APEX1 (Hs00172396_m1), CENPB (Hs00374196_s1), EIF5A (Hs00744729_s1), HMGA1 (Hs00852949_g1), PES1 (Hs00897727_g1), POLR2E (Hs00267554_m1), POLR2K (Hs01562397_m1), PPP1R14B (Hs02598738_g1), RHOC (Hs00237129_m1), TGM2 (Hs01096681_m1), EPN1 (Hs00203391_m1), IMPDH1 (HS04190080_GH), CPTP (Hs00257998_s1), SERPINE1 (Hs00167155_m1), VAMP3 (Hs00933164_m1), PPIL1 (Hs01080974_m1), TPP2 (Hs00162879_m1), RFC3 (Hs01082404_m1), UMPS (HS00923517_m1) and GAPDH control (Hs00266705_g1). The relative expression of each target gene was normalized to the level of GAPDH except in the analysis post biotin pull-down. Statistical analysis was performed using Student’s *t* test.

### Luciferase reporter assay

293T cells were co-transfected in triplicates in 96 well plates, 24 h post plating, with 50 ng of each luciferase reporter clones and 20 nM of either siNT1 or siGGGGGC using Lipofectamine 2000 (Thermofisher). The control plasmid, pMirTarget (#PS100062); PES1 human clone (#SC205900, NM_014303); POLR2K human clone (#SC208896; NM_005034); POLR2E human 3UTR clone (#CW306737, NM_00131623); EIF5A human 3UTR clone (#SC208525, NM_001143760); Mutant 3′ UTR clones for PES1 (#CW306514), POLR2K (#CW306515), POLR2E (#CW306738), and EIF5A (#CW306739) were all purchased from Origene. Mutant plasmids were generated by synthesizing 3′ UTR sequences in which the three GCCCCC seed matches in the 3′ UTR of PES1, one in 3′ UTR of POLR2K, three in POLR2E 3′ UTR and 5 seed matches in EIF5A 3′ UTR was each changed to TGCAAA, and this altered sequence was each inserted into the pmiRTarget construct by Origene. After 48 h, transfection efficiency was determined by measuring Red Fluorescence Protein (RFP) expression of the luciferase reporter plasmid followed by measurement of luciferase activity after lysing cells with Bright-Glo Luciferase Assay System (Promega #E2610), both using Biotek Cytation 5 plate reader. Data are shown as relative luciferase activity normalized to RFP expression.

### Pull-down of biotinylated siRNAs

HeyA8 cells (1.6 × 10^6^) were transfected in 10 cm dishes in biological replicates with 50 nM biotinylated (Bi)-siNT1 or Bi-siGGGGGC (duplexes with a biotin coupled to the 3′ end of the antisense strand in each case) as described in^28^. Cells were harvested 20 h after transfection and pelleted at 500×*g*. Cell pellet was washed twice in cold PBS and then resuspended in lysis buffer plus [2.5 mg/ml Ficoll PM400, 7.5 mg/ml Ficoll PM70, 250 μg/ml Dextran Sulfate (Sigma #D8906), 200 U/ml RNaseOUT™ and 100 U/ml SUPERase.In™ (Life Technologies)] and incubated on ice for 20 min. The cytoplasmic lysate was extracted at 5000×*g* for 5 min. Streptavidin magnetic beads (Dynabeads M-280; Life Technologies) were blocked for 2 h at 4 °C in lysis buffer (20 mM Tris–HCl (pH 7), 100 mM KCl, 5 mM MgCl2, 25 mM EDTA, 0.3% NP-40 with proteinase Inhibitor cocktail) containing 1 mg/ml yeast tRNA and 1 mg/ml BSA (both from Ambion) and then washed with lysis buffer. The cytoplasmic lysates were incubated with beads for 4 h at 4 °C. The pull-down RNA (RNA bound to the beads) or Input RNA (10% of lysate) were isolated using Trizol LS reagent (Invitrogen). The level of the 5 siGGGGGC target mRNAs (EIF5A, PES1, POLR2E, HMGA1, POLR2K), 5 control mRNAs (VAMP3, PPIL1, RFC3, TPP2, UMPS) and a housekeeping gene (GAPDH) was quantified using real-time PCR. The expression level in both Bi-siNT1 and Bi-siGGGGGC pull-down RNA was normalized to the respective input RNA levels.

### Empirical cumulative distribution function (eCDF) plot

RNA-seq data of HeyA8 cells transfected with siNT1, siGGGGGC or siGGCAGU (24 h) or of HeyA8 and PC3 cells transfected with siNT1 or siGGGGGC (24 and 48 h combined) were used. In order to determine if the mRNAs are regulated based on presence of GCCCCC (seed match for siGGGGGC) or ACUGCC (seed match for siGGCAGU) seeds in their 3′ UTR, a custom R script was used. The R script takes as input a list of genes containing the seed and a list of genes not-containing the seed in their 3′ UTR, in each case, as well as a table of logFC expression for those genes upon siGGGGGC or siGGCAGU over-expression. This script then generates the eCDF plot for the logFC expression data for each gene set. The custom scripts and the input data files for the eCDF plots are available in Code Ocean at https://codeocean.com/capsule/5453168/tree for siGGGGGC and at https://codeocean.com/capsule/8338685/tree for siGGCAGU (for HeyA8 cells) and at https://codeocean.com/capsule/9825010/tree for PC3 cells. Kolmogorov–Smirnov (K–S) two-sample test (alternative hypothesis = “greater”) was used to compare different probability distributions shown in the eCDF plots.

### Western blot

Western blot for was performed as previously described^13^. The membranes were blocked in 5% non-fat dry milk in 0.1% TBS/Tween-20 and incubated in primary antibodies overnight at 4 °C. Both primary and secondary antibodies were diluted in the above blocking buffer as follows: anti-EIF5A (1:1000), anti-PES1 (1:1000), anti-POLR2E (1:500), anti-β-actin IgG HRP (1:5000), anti-rabbit IgG HRP (1:5000), anti-mouse IgG HRP (1:5000). Protein bands were detected using the SuperSignal™ West Dura Extended Duration Substrate (Thermo Fisher Scientific #34076). Densitometry of the western blot bands was done using the Syngene software package GeneTool. Uncropped blots are shown in Fig. S2.

### Statistical analyses

IncuCyte experiments were performed in triplicates and the data were expressed as mean ± standard error, continuous data were compared using Student's *t* tests for two independent groups. Two-way ANOVA was performed using the Stata1C software for evaluating continuous outcomes over time, choosing one component for the treatment condition as primary interest and a second component for time as a categorical variable. Multinomial mixed effects regression model to analyze 6mer seed compositions. A novel approach for the analysis of sequence logo data using a generalized linear mixed model for multinomially distributed was developed^29^. Sequence logos (generated with Weblogo (http://weblogo.berkeley.edu/logo.cgi) are a graphical representation of the nucleotides in each of the 6 positions of the 6mer seed, but they only permit a visual representation. Multinomial mixed models provide estimates of the relative differences in nucleotide composition between groups and positions as well as a statistical test of such differences. In this framework, we represent each 6mer seed as 6 “repeated measures” and assume that nucleotides follow a multinomial distribution at each position. A multinomial mixed effects model is then fitted with the nucleotide (A, C, G, U) as the outcome; group, position and their interaction as the fixed effects; and 6mer id as the random effect. Correlation between the 6 positions is accounted for using “unstructured” covariance structure. Because the toxic seeds are dominated by Gs we compare the odds of either A, U, or C to that of G in each seed position and obtained odds ratio (OR) estimates comparing groups and positions. An interaction term allows us to determine whether the nucleotide composition differences between groups vary by position. The multinomial mixed models were fitted using PROC GLIMMIX in SAS v. 9.4.

### Importance

We recently identified a robust form of cell death that is induced by expression of toxic small RNAs carrying a sequence that can selectively kill cancer cells: the 6mer seed. We performed a high throughput screen with siRNAs containing all possible 4096 6mers in a neutral backbone and found that the most toxic 6mer seed consensus was GGGGGC. While previously we described this death mechanism as an off-target effect, we now demonstrate that it is in fact an on-target effect by showing that an siRNA carrying GGGGGC seed, acts like a miRNA, and kills cancer cells by directly targeting the seed matches in the 3′ UTR of several essential survival genes. Our data suggests that this mechanism arose through co-evolution of miRNAs and seed matches in 3′ UTRs of mRNAs to prevent highly abundant miRNAs from targeting survival genes and inducing cell death in differentiated cells.

## Supplementary Information

Supplementary Movie S1.

Supplementary Movie S2.

Supplementary Movie S3.

Supplementary Information.

Supplementary Table S1.

Supplementary Table S2.

## Data Availability

RNA Seq data were deposited at GEO under accession numbers GSE164289 (HeyA8 cells) and GSE196839 (HeyA8 and PC3 cells, used in Fig. S1).

## References

[1] Lewis BP, Shih IH, Jones-Rhoades MW, Bartel DP, Burge CB (2003). Prediction of mammalian microRNA targets. Cell.

[2] Lai EC (2002). Micro RNAs are complementary to 3′ UTR sequence motifs that mediate negative post-transcriptional regulation. Nat. Genet..

[3] Wang Y, Sheng G, Juranek S, Tuschl T, Patel DJ (2008). Structure of the guide-strand-containing argonaute silencing complex. Nature.

[4] Alles J (2019). An estimate of the total number of true human miRNAs. Nucleic Acids Res..

[5] Yan M (2018). miR-146b promotes cell proliferation and increases chemosensitivity, but attenuates cell migration and invasion via FBXL10 in ovarian cancer. Cell Death Dis..

[6] Svoronos AA, Engelman DM, Slack FJ (2016). OncomiR or tumor suppressor? The duplicity of microRNAs in cancer. Cancer Res..

[7] Chan JK (2014). The inhibition of miR-21 promotes apoptosis and chemosensitivity in ovarian cancer. Gynecol. Oncol..

[8] Hadji A (2014). Death induced by CD95 or CD95 ligand elimination. Cell Rep..

[9] Putzbach W (2018). DISE—A seed dependent RNAi off-target effect that kills cancer cells. Trends Cancer.

[10] Putzbach W (2017). Many si/shRNAs can kill cancer cells by targeting multiple survival genes through an off-target mechanism. Elife.

[11] Haluck-Kangas A (2021). DISE/6mer seed toxicity—A powerful anti-cancer mechanism with implications for other diseases. J. Exp. Clin. Cancer Res..

[12] Corbin JM (2021). Seed-mediated RNA interference of androgen signaling and survival networks induces cell death in prostate cancer cells. Mol. Ther. Nucleic Acids.

[13] Gao QQ (2018). 6mer seed toxicity in tumor suppressive miRNAs. Nat. Commun..

[14] Stark A, Brennecke J, Bushati N, Russell RB, Cohen SM (2005). Animal MicroRNAs confer robustness to gene expression and have a significant impact on 3′UTR evolution. Cell.

[15] Murmann AE (2019). 6mer seed toxicity in viral microRNAs. iScience.

[16] Slabakova E, Culig Z, Remsik J, Soucek K (2017). Alternative mechanisms of miR-34a regulation in cancer. Cell Death Dis..

[17] Murmann AE (2017). Induction of DISE in ovarian cancer cells in vivo. Oncotarget.

[18] Murmann AE (2018). Small interfering RNAs based on huntingtin trinucleotide repeats are highly toxic to cancer cells. EMBO Rep..

[19] Putzbach W (2018). CD95/Fas ligand mRNA is toxic to cells. Elife.

[20] Patel M (2021). The ratio of toxic-to-nontoxic microRNAs predicts platinum sensitivity in ovarian cancer. Cancer Res..

[21] van Dongen S, Abreu-Goodger C, Enright AJ (2008). Detecting microRNA binding and siRNA off-target effects from expression data. Nat. Methods.

[22] Beg MS (2017). Phase I study of MRX34, a liposomal miR-34a mimic, administered twice weekly in patients with advanced solid tumors. Investig. New Drugs.

[23] Bolger AM, Lohse M, Usadel B (2014). Trimmomatic: A flexible trimmer for Illumina sequence data. Bioinformatics.

[24] Kim D (2013). TopHat2: Accurate alignment of transcriptomes in the presence of insertions, deletions and gene fusions. Genome Biol..

[25] Dobin A (2013). STAR: Ultrafast universal RNA-seq aligner. Bioinformatics.

[26] Anders S, Pyl PT, Huber W (2015). HTSeq—A Python framework to work with high-throughput sequencing data. Bioinformatics.

[27] Robinson MD, McCarthy DJ, Smyth GK (2010). edgeR: A bioconductor package for differential expression analysis of digital gene expression data. Bioinformatics.

[28] Tan SM, Lieberman J (2016). Capture and identification of miRNA targets by biotin pulldown and RNA-seq. Methods Mol. Biol..

[29] Bartom, E. T. *et al.* SPOROS: A pipeline to analyze DISE/6mer seed toxicity. *PLOS Comp. Biol.*, in press (2022). 10.1371/journal.pcbi.1010022 PMC9004739 35358200

