# Supplementary material for: Identification of the toxic 6mer seed consensus for human cancer cells
Source: Sci Rep. 2022 Mar 24;12:5130. doi: 10.1038/s41598-022-09051-w (PMC8948288; doi:10.1038/s41598-022-09051-w)
Supplement: Supplementary file 4 — Supplementary Information. [file 41598_2022_9051_MOESM4_ESM.pdf]

Supplementary Information

Identification of the toxic 6mer seed consensus for human cancer cells

Monal Patel, Elizabeth T. Bartom, Bidur Paudel, Masha Kocherginsky, Kaitlyn L. O'Shea, Andrea E. Murmann and Marcus E. Peter

Supplementary Table S1: Results of the screen of 4096 seed containing siRNAs on three human and three murine cells.
Cells are color-coded: Most toxic seeds are in red and least toxic ones in green.

Supplementary Table S2: Fold change of all genes substantially expressed in HeyA8 cells transfected with siGGGGGC or siGGCAGU, compared to siNT1.
Analysis of a data set from an RNA Seq analysis of HeyA8 cells transfected with 10 nM of either siGGGGGC or siGGCAGU, both in comparison to 10nM siNT1. Data are ranked according to highest Log2 fold downregulation. The top ten most downregulated transcripts are highlighted in green, the bottom ones in yellow. Amongst the top ten downregulated genes, the ones labeled in red also belong to the curated list of survival genes.

Supplementary Movies S1-S3: HeyA8 cells transfected with 10 nM siNT1 (Movie S1), siGGCAGU (Movie S2), or siGGGGGC (Movie S3).

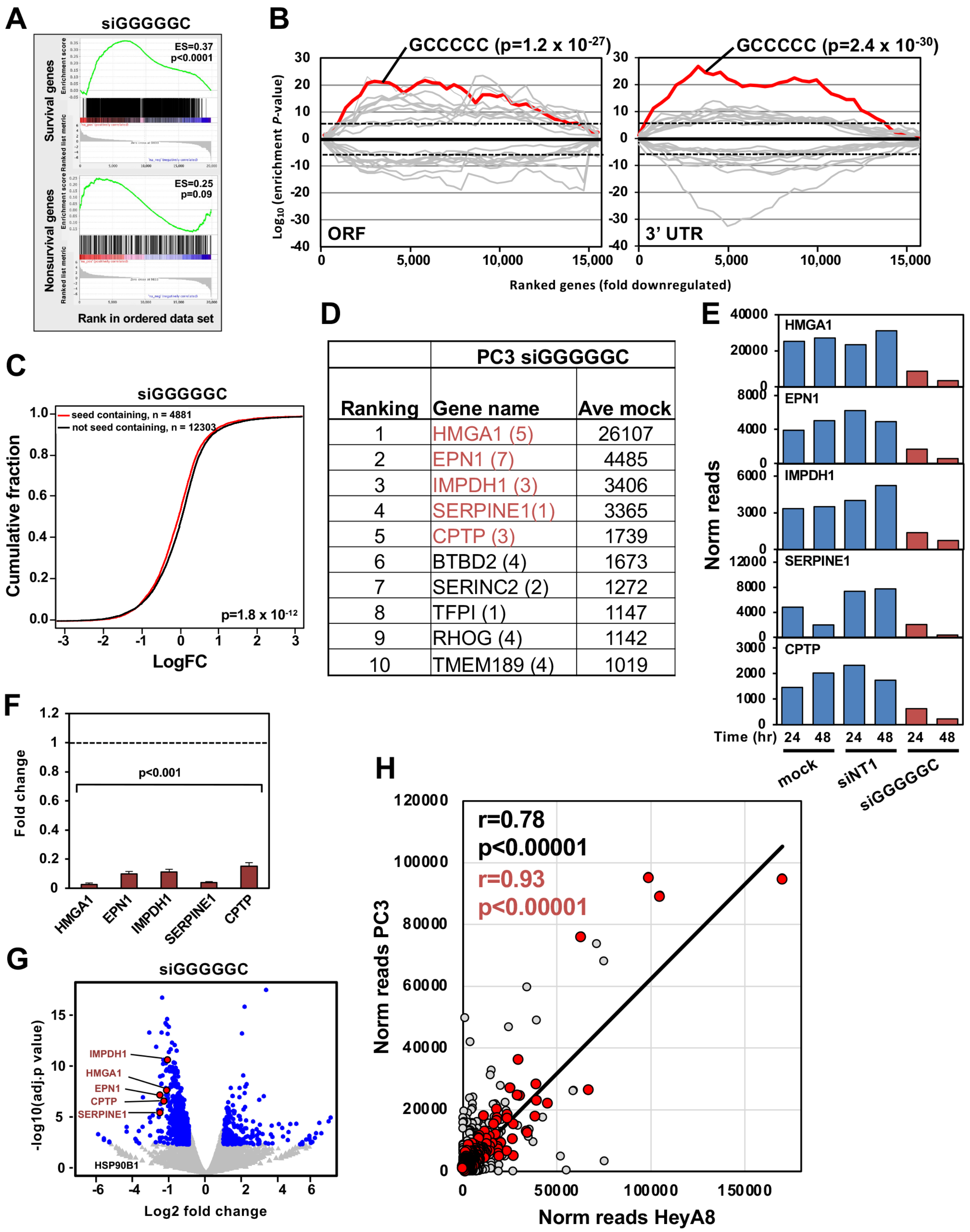

	PC3 siGGGGGC	
Ranking	Gene name	Ave mock
1	HMGA1 (5)	26107
2	EPN1 (7)	4485
3	IMPDH1 (3)	3406
4	SERPINE1(1)	3365
5	CPTP (3)	1739
6	BTBD2 (4)	1673
7	SERINC2 (2)	1272
8	TFPI (1)	1147
9	RHOG (4)	1142
10	TMEM189 (4)	1019

Supplementary Figure S1: Identification of genes in PC3 cells targeted by siGGGGGC.
(A) Gene set enrichment analysis for a group of 1846 survival genes (top panel) and 416 non-survival genes (bottom panel) after transfecting PC3 cells with 10 nM siGGGGGC (24 and 48 hrs time points combined). siNT1 served as a control. p-values indicate significance of enrichment; the enrichment score (ES) is shown.
(B) Sylamer analysis (6mers) for the list of open reading frames (ORFs; left) and 3‘ UTRs (right) of mRNAs in cells in (A), sorted from down-regulated to up-regulated compared to cells transfected with siNT1. The 10 most

and ten least enriched seeds are shown. Enrichment of the GCCCCC sequence is shown in red, other sequences are in grey. The stippled line corresponds to a p-value threshold of 0.05 after Bonferroni correction for the number of words tested (4096). Bonferroni-adjusted p-values are shown.

(C) eCDF plot of mRNAs in RNA isolated from cells in (A) and containing a unique 6mer seed match for siGGGGGC in their 3’ UTR vs those that do not. The p-values were calculated using a two-sample Kolmogorov-Smirnov (K-S) test (alternative hypothesis= “greater”).

(D) Top ten most downregulated genes in PC3 cells transfected with siGGGGGC, ranked according to highest average read number on mock treated cells. The number of GCCCCC seed matches is given in brackets. The top 5 most abundant genes analyzed in E are shown in red.

(E) Read number of the genes labeled red in D in PC3 cells lipid treated (mock), or transfected with either siNT1 or siGGGGGC for 24 or 48 hrs.

(F) Real time PCR validation of the 5 genes in E. Gene expression levels were normalized to GAPDH and to cells transfected with siNT1 (stippled line). Each bar represents ± SD of three replicates. Students t-test was used to calculate p-values.

(G) Volcano plot of deregulated genes in PC3 cells transfected with 10 nM siGGGGGC vs. siNT1. Red dots indicate the top 5 genes in D that have one or more predicted GCCCCC seed matches in their 3' UTR. Grey dots indicate all differentially expressed genes (with a log2 fold change <6 and >-6), blue dots indicate genes with log2 fold change>1 and FDRAdjpValue<0.05.

(H) Regression analysis of all genes expressed at 1000 or more normCounts and containing at least one GCCCCC 6mer in their 3' UTR in either HeyA8 or PC3 cells (mock treated). Grey dots, all genes; red dots, survival genes. Pearson correlation coefficients (r) and p-values are given for both groups in matching colors.

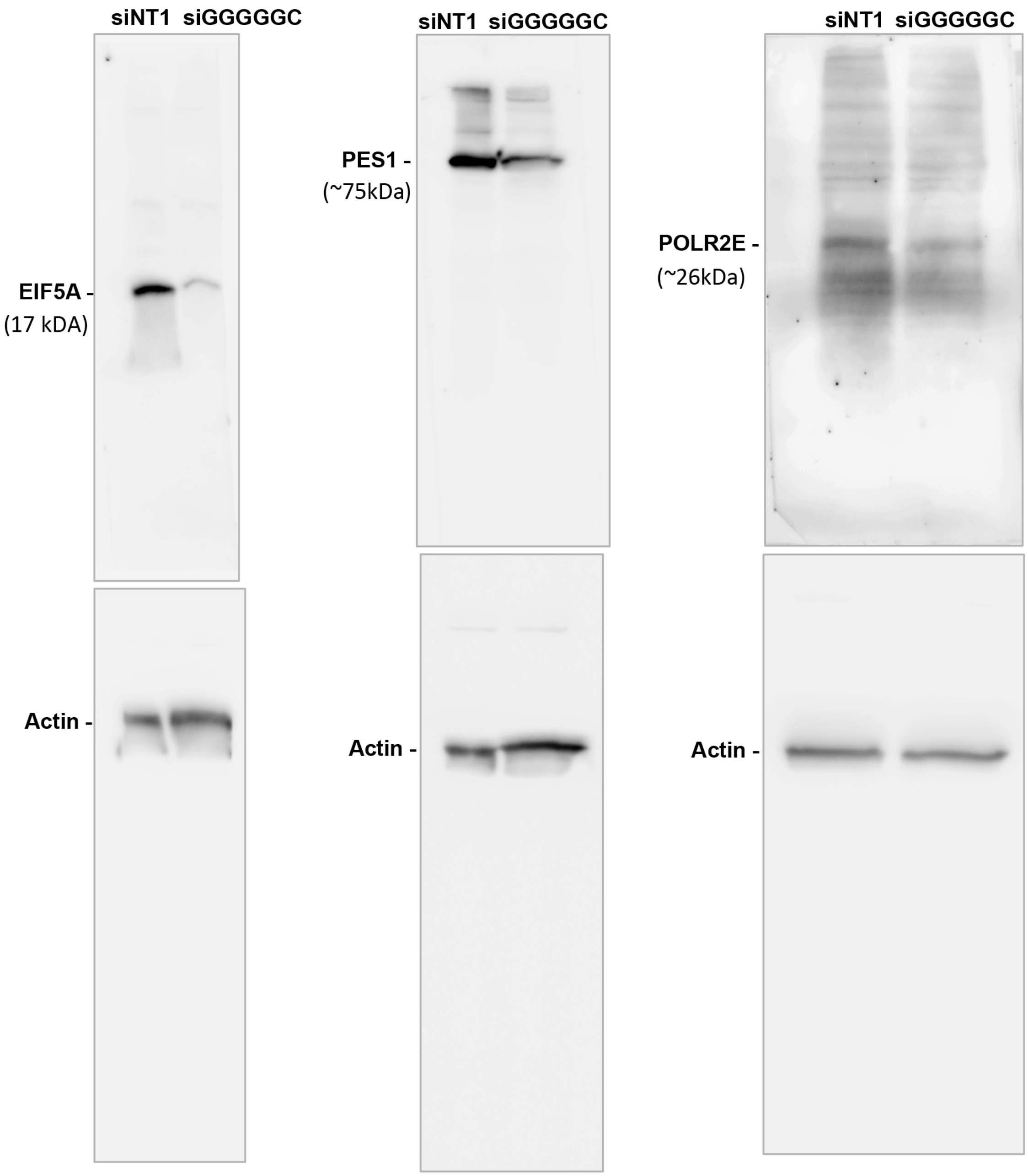

Supplementary Figure S2: Uncropped Western blots of Figure 4F.
